# Thermodynamic, Kinetic, and UV–Vis/CD Spectroelectrochemical Studies on Interaction and Electron Transfer Between Glucose Oxidase and Ferrocene Carboxylic Acid

**DOI:** 10.3390/molecules31010102

**Published:** 2025-12-26

**Authors:** Luis Gabriel Talavera-Contreras, Marisela Cruz-Ramírez, Juan Pablo F. Rebolledo-Chávez, Janet Ocampo-Hernández, Gilberto Rocha-Ortiz, Luis Ortiz-Frade

**Affiliations:** 1 Departamento de Electroquímica, Centro de Investigación y Desarrollo Tecnológico en Electroquímica, S. C. Parque Tecnológico Querétaro, Sanfandila, Pedro de Escobedo 76703, Querétaro, Mexico; ltalavera@cideteq.mx (L.G.T.-C.);; 2 Colegio de Bachilleres, Universidad Autónoma de Querétaro, Campus San Juan del Río, Calle Corregidora No. 4, Colonia Centro, San Juan del Río 76800, Querétaro, Mexico; 3 División de Química y Energías Renovables, Universidad Tecnológica de San Juan del Río, Avenida La Palma No. 125. Vista Hermosa, San Juan del Río 76826, Querétaro, Mexico; jprebolledoc@utsjr.edu.mx

**Keywords:** glucose oxidase, ferrocene carboxylic acid, molecular interaction, redox mediator, spectroelectrochemistry, homogeneous electron transfer

## Abstract

In this research, we investigate the interaction between the redox mediator ferrocene carboxylic acid (Fc-COOH) and glucose oxidase (GOD) in order to determine the thermodynamics parameters K_int_, ΔG_int_, ΔH_int,_ and ΔS_int_ using simple UV–visible experiments at different temperatures. Positive values of ΔH_int_, ΔS_int_, together with a negative value of ΔG_int_ indicate an entropy-driven hydrophobic interaction typical of spontaneous association processes. The homogeneous electron transfer rate constants between the oxidized organometallic mediator and the reduced enzyme (k_s_), along with their activation parameters (ΔG_ET_^≠^, ΔH_ET_^≠^ and ΔS_ET_^≠^), were calculated using data obtained from foot of the wave analysis (FOWA) of cyclic voltammetry experiments performed at variable temperature. According to transition state theory, the obtained parameters indicate a low activation enthalpy that reflects minimal energetic requirements for electron transfer, while the large negative activation entropy suggests the formation of an ordered transition state. The positive activation free energy falls within the expected range for biological electron transfer processes. Variable temperature cyclic voltammetry experiments of ferrocene carboxylic acid (Fc-COOH) were also performed. The obtained ΔG°, ΔH°, and ΔS° parameters indicate strong stabilization of the redox pair, consistent with a small difference in solvation energy. Circular dichroism, UV–vis spectroscopy, and combined CD and UV–Vis Spectroelectrochemistry measurements performed during redox mediation demonstrate that no significant structural alterations occur in either the enzyme or the redox mediator before or during the electron transfer processes.

## 1. Introduction

During the last few decades, glucose oxidase (GOD) has played a central role in the development of electrochemical biosensors for the monitoring and diagnosis of diabetes mellitus (DM). Although numerous studies have addressed glucose quantification using GOD, this enzyme remains of considerable interest for the design of high-performance biofuel cells, artificial pancreas devices, and as a model system for analyzing electron transfer processes in redox proteins [1]. GOD is a flavoenzyme oxidoreductase with an approximate molecular mass of 160 KDa and contains flavin adenine dinucleotide (FAD) as a tightly bound cofactor. FAD is located within a hydrophobic cavity of the protein and mediates reversible redox transformations between its oxidized (FAD) and reduced (FADH_2_) forms. This cofactor acts as an acceptor of two electrons and two protons during the oxidation of β−D−glucose to D−glucono−δ−lactone, as illustrated by the following Equations (1) and (2) [2].

(1) $$\beta -D-glucose+GOD\;\left(FAD\right)\to \;D-glucono-\delta -lactone+GOD\;\left(FADH_{2}\right)\;$$

(2) $$GOD\left(FADH_{2}\right)+O_{2}\;\to GOD\;\left(FAD\right)+H_{2}O_{2}$$

Because the active site of glucose oxidase (FAD) is deeply embedded within the protein matrix, direct electron transfer to an electrode is kinetically hindered, and no significant current is typically observed for biosensing or energy generation applications. Numerous strategies have been explored to enhance electron transfer, including the use of carbon nanotubes [3,4], platinum group nanoparticles [5,6], reconstructed or immobilized GOD constructs [7,8,9], coordination compounds, and organometallic redox mediators [10,11,12]. The latter approach offers distinct advantages, such as straightforward synthesis, efficient regeneration of reduced GOD, and precise control over the redox active species.

In redox mediated systems, the homogeneous electron transfer rate constant ($k_{s}$) between the oxidized mediator Med (ox) and the reduced GOD (FADH_2_) constitutes the key step governing current enhancement. It has been proposed that k_s_ follows the Marcus cross relation (Equation (3), [13]), where k_11_ and k_22_ represent the self-exchange rate constants of the participating redox couples. The term *f*_12_ is a statistical factor relating k_11_ and k_22_ to the collision frequency of the reactants, typically close to unity. The equilibrium electron transfer constant, K_ET_, is obtained by equating the Nerst equations of the couples Med(Red)/Med(Ox) and GOD(FAD)/GOD(FADH_2_) and depends on the difference in redox potentials for the exchange of **n** electrons, as shown in Equation (4).

(3) $$k_{s}=\sqrt{f_{12}k_{11}k_{22}K_{ET}}\;$$

(4) $$K_{ET}=10^{\frac{{\left\lfloor E_{\frac{Med\left(Ox\right)}{Med\left(Red\right)}}^{0}-E_{\frac{GOD\left(FAD\right)}{GOD\left(FADH_{2}\right)}}^{0}\right\rfloor }^{n}}{0.059}}$$

However, the interaction between the enzyme and the redox mediator prior to the electron transfer is not accounted for in this model. Moreover, neither the activation parameters nor the molecular structural changes experienced for GOD during electron transfer have been explored. These factors may significantly influence glucose sensing performance and could contribute to the development of more accurate predictive models.

Ferrocene derivates are utilized as mediators in glucose sensors owing to their chemical stability, lower redox potential, biocompatibility, and reversible electrochemical characteristics. These properties facilitate the effective mediation of glucose oxidation [14,15]. Several ferrocene derivates have been extensively studied as a redox mediator for GOD, where their homogeneous electron transfer rate constants (k_s_) were determined using the Nicholson and Shain methods [16]. Notably, Fc-1, 1-CH_3_, Fc-H, Fc-CH=CH_2_, and Fc-COOH exhibited ks values of 7.76 × 10^4^, 2.57 × 10^4^, 3.01 × 10^4^, and 2.01 × 10^5^ L/(mol·s), respectively. It is evident that Fc-COOH achieved the highest k_s_ among the tested ferrocene derivates where a strong interaction with GOD is proposed [17,18,19]. Additionally, Fc-COOH has shown reproducibility tests indicate that biosensors with this redox mediator sustain consistent response signals across multiple cycles with a deviation of less than 5% in peak current values [20]. The already presented properties make Fc-COOH a promising candidate, not only for biosensors, but also for other applications such as biofuel cells [21]. However, the interaction between Fc-COOH with GOD prior to electron transfer have not been demonstrated. Additionally, changes in molecular structural of GOD during electron transfer has not been explored. Both effects in combination should be considered in models that describe performance glucose biosensing and in other second-generation biosensors. Hence in this research, we investigate the molecular interaction between ferrocene carboxylic acid and glucose oxidase (GOD), using simple UV–visible experiments at different temperatures, to the obtain the thermodynamic parameter (K_int_, ΔG_int_, ΔH_int_ and ΔS_int_). Furthermore, the homogeneous electron transfer rate constants between the oxidized organometallic mediator and the reduced enzyme (k_s_) with their activation parameters (ΔG_ET_^≠^, ΔH_ET_^≠^ and ΔS_ET_^≠^), were obtained using a foot of the wave analysis (FOWA) from cyclic voltammetry at variable temperature. Cyclic voltammetry of ferrocene carboxylic acid (Fc-COOH) at variable temperature was carried out to study the stability of the redox pair trough ΔG°, ΔH°, and ΔS° parameters. We conducted circular dichroism, UV–vis spectroscopy, as well as CD and UV–Vis spectroelectrochemistry measurements during the redox mediation. This research includes the application of CD and UV–Vis spectroelectrochemistry for the first time to evaluate potential structural modifications in the protein during the electron transfer process.

## 2. Results and Discussion

### 2.1. Calculation of the Equilibrium Constant and Thermodynamic Parameters for the Interaction Between GOD and Ferrocene Carboxylic Acid

**Figure 1:**
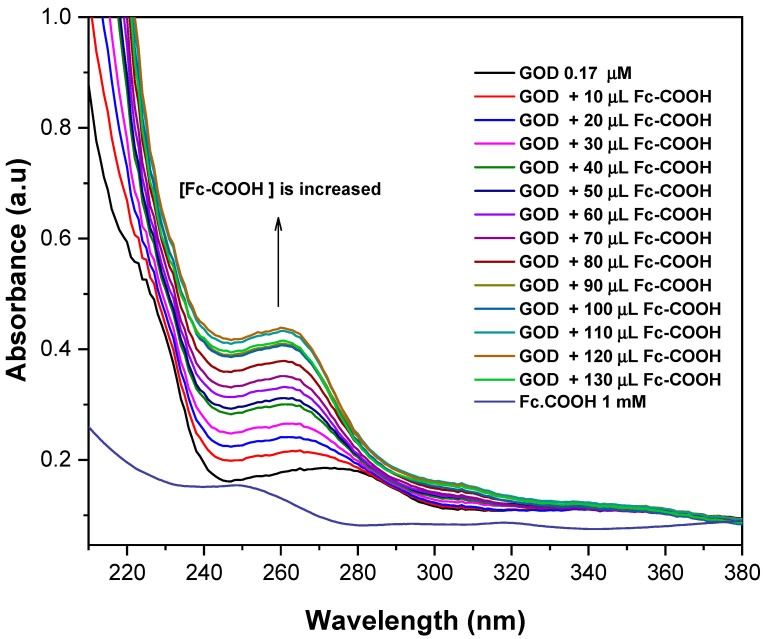
UV–Vis spectra of 0.17 mM GOD in PBS 0.1M (pH = 7.2) in the presence of Fc-COOH 1 mM at 25 °C.

Figure 1 shows the UV–Vis spectra of GOD (0.17 mM) recorded in the presence of increasing concentrations of Fc-COOH. The main electronic transition of the enzyme, initially observed at 275 nm (0.087 a.u.), progressively shifts to 260 nm accompanied by an increase in absorbance intensity (0.435 a.u.). This spectral behavior is consistent with the formation of an electron donor–acceptor complex mediated by weak noncovalent interactions. Similar responses have been previously reported for interactions between the tyrosine residues in GOD and various molecular probes; in the present case, the observed changes are attributed to the interaction between GOD and Fc-COOH [22,23].

The interaction constant (K_int_) was determined from the change in absorbance at 260 nm, where the formation of the electron donor–acceptor complex is most evident. The analysis was performed according to the Benesi–Hilderbrand model, (Equation (5)). In this equation, A_0_ represents the initial absorbance of GOD, A is the absorbance measured in the presence of Fc-COOH and A_max_ corresponds to the maximum absorbance associated with the formation of donor–acceptor complex under conditions of excess Fc-COOH [24,25].

**Figure 2:**
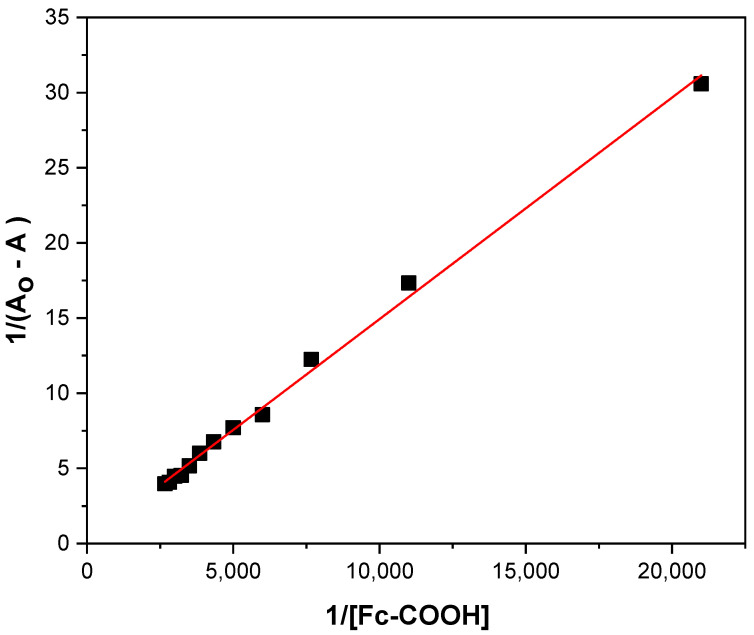
Benesi–Hilderbrand equation at 260 nm for 0.17 mM GOD enzyme and additions of 1 × 10^−3^ M Fc-COOH in PBS 0.1 M (pH = 7.2) with temperature control at 25 °C.

A Benesi–Hilderbrand plot of $\frac{1}{\left(A_{max}-A_{0}\right)}\;vs.\;[Fc-COOH]$ at 260 nm yields a linear relationship, with an intercept of $\frac{1}{\left(A_{max}-A_{0}\right)}\;$ and a slope of $\frac{1}{K_{int}\left(A_{max}-A_{0}\right)}$ (Figure 2). From the ratio of the slope to the interception, a K_int_ value of 125 ± 10 mol^−1^ was obtained. Control electronic spectra of Fc-COOH, (Figure S1), as well as experiments performed in the absence of GOD (Figure S2), confirm that no spectral interference affects the determination of the interaction constant (K_int_).

(5) $$\frac{1}{\left(A-A_{0}\right)}=\frac{1}{K_{int}\left(A_{max}-A_{0}\right)[C]}+\frac{1}{\left(A_{max}-A_{0}\right)}$$

To determine the thermodynamic parameters associated with the interaction between GOD and Fc-COOH, the equilibrium constant (K_int_) was evaluated at different temperatures. Assuming that the enthalpy change (ΔH) remains approximately constant within the temperature range studied, the entropy (ΔS_int_) and free energy (ΔG_int_) values were obtained using the Van’t Hoof equation (Equation (6). The corresponding thermodynamic parameters were then calculated from the relationship ΔG_int_ = ΔH_int_ − TΔS_int_, (Table 1 and Figure S3).

(6) $$LnK_{int}=-\frac{\Delta H_{int}}{RT}+\frac{\Delta S_{int}}{R}\;$$

According to the literature, the positive values of ΔH_int_ and ΔS_int_ obtained are indicative of a hydrophobic interaction. This behavior can be rationalized by considering that hydrophobic amino acids side chains, initially exposed to the solvent, become partially shielded upon binding of the mediator. This process releases ordered water molecules from the hydration shell into the bulk solution, thereby increasing the overall disorder or the system. The negative ΔG_int_ values confirm the spontaneous nature of this entropy-driven interaction process [26].

Additionally, UV–Vis experiments performed with bovine serum albumin (BSA) under the same conditions used for GOD revealed no evidence of electron donor–acceptor complex formation with Fc-COOH. This result indicates that Fc-COOH has no measurable affinity for BSA (Figure S4).

The value of interaction constant K_int_ obtained (125 ± 10 L/mol) reveals a significant affinity between Fc-COOH and GOD, consistent with the level of pre-association required to facilitate fast electron-transfer processes without perturbing the protein structure. The positive ΔH_int_ and ΔS_int_ values clearly indicate a hydrophobic, entropy-driven interaction, while the negative free energy (ΔGi_nt_ = −31 kJ/mol) confirms that the association is spontaneous and thermodynamically favorable, which is consistent with the behavior expected for efficient redox mediators approaching buried enzyme cofactors. Table 1 summarizes the equilibrium constant and thermodynamic parameters for the interaction between GOD and Fc-COOH. However, no similar data for GOD with other redox mediators are reported in the literature, opening a new door for research in this area. In future work we will study the molecular interaction between GOD and other ferrocene derivatives to propose a model that consider this effect in glucose biosensing performance.

### 2.2. Effect of Temperature on Redox Potential

Cyclic voltammograms recorded at 25 °C for 1 × 10^−3^ M ferrocene monocarboxylic acid (Fc-COOH) in PBS (pH = 7.2) at various scan rates are shown in Figure S5. The anodic and cathodic peak separation (59 mV to 60 mV) is consistent with a reversible one electron redox process, yielding a formal potential of 305 mV vs. Ag/AgCl. The ratio of cathodic to anodic peak currents (i_pc_/i_pa_) remains close to unity, confirming the reversibility of the Fc-COOH redox couple.

Temperature dependent measurements (25 °C to 50 °C) revealed the expected shift in E°, as shown in Figure S6. A plot of E° vs. temperature (T) exhibits a linear trend described by E°= −0.0003287T + 0.01181 (r = 0.98), presented in Figure S7. Using the slope (ΔE°/ΔT) and the formal redox potential (E°), the standard thermodynamic parameters ΔH° and ΔS° were calculated according to Equations (7) and (8). The Gibbs free energy, ΔG°, was subsequently obtained from ΔG° = ΔH − TΔS°.

The resulting thermodynamics parameters ΔG°= −28.7 KJ/mol, ΔH° = 1228 KJ/mol, and ΔS° = −9.6 KJ/mol have not been previously reported for this redox mediator. These values indicate a high stabilization for the redox pair due to small difference in solvation energy between the oxidized and reduced forms, accompanied by an increase in solvent orientation. Such behavior is consistent with an outer sphere electron transfer mechanism and agrees with the behavior reported [Fe(CN)_6_]^4−^/[Fe(CN)_6_]^3−^ [27,28].

(7) $$∆S{}^{\circ}=-nF{\left(\frac{\partial E{}^{\circ}}{\partial T}\right)}_{p}\;$$

(8) $$∆H{}^{\circ}=-nFE{}^{\circ}+nFT{\left(\frac{\partial E{}^{\circ}}{\partial T}\right)}_{p}$$

### 2.3. Calculation of the Homogeneous Electron Transfer Rate Constant GOD-Mediator

To evaluate the homogeneous electron transfer rate constant between GOD and Fc-COOH, cyclic voltammetry experiments were carried out at a scan rate of 1 mV/s for 1 × 10^−3^ M Fc-COOH in PBS (pH = 7.2) at 25 °C, both in the absence and in the presence of 0.05 M glucose and 1.8 mM GOD.

**Figure 3:**
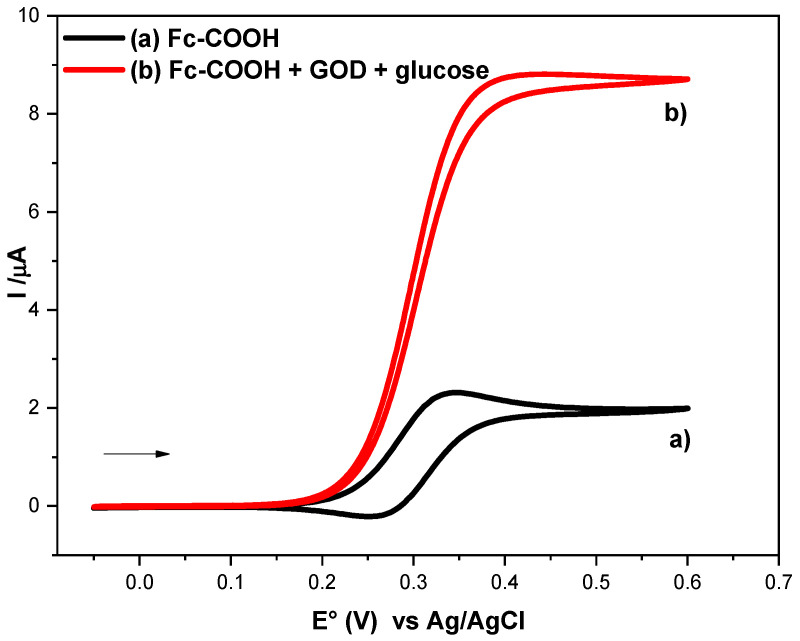
Cyclic voltammograms of 1 × 10^−3^ M Fc-COOH in PBS 0.1M (pH = 7.2) in the presence of 0.05 M glucose in the absence (a) and in the presence (b) of 1.8 mM GOD enzyme at 1 mVs^−1^.

As shown in Figure 3 (a), in the absence of the enzyme, a reversible diffusion-controlled process is observed, characterized by a peak current (i_p_°). In contrast, upon addition of GOD (Figure 3, (b)), a catalytic current plateau (i_k_) emerges, which is indicative of an EiC’ mechanism, involving the following steps (Equations (9)–(11)):

(9) $$glucose+GOD\left(FAD\right)\to gluconolactone+GOD\left(FADH_{2}\right)$$

(10) $$GOD\left(FADH_{2}\right)+2FcCOOH^{+}\;\overset{ks}{\to }\;GOD\left(FAD\right)+2Fc-COOH+2H^{+}$$

(11) $$2FcCOOH⇄2FcCOOH^{+}+2e^{-}$$

The rate constant (*k_s_*) in step II corresponds to the homogeneous electron transfer between ferricenium (Fc-COOH^+^) and the reduced form of the enzyme GOD (FADH_2_). This step constitutes the key process governing redox mediation. Although *k_s_* can, in principle, be calculated using the method reported by Nicholson and Shain [16], this approach presents several limitations, including the requirement of prior knowledge of diffusion coefficient and the assumption that no additional coupled chemical reactions occur. Therefore, the foot to the wave analysis (FOWA) method was employed in this study to determine *k_s_* according to Equation (12) [29,30,31].

(12) $$\frac{i_{k}}{i_{p}^{0}}=\frac{2.24\sqrt{\frac{RT}{Fv}2k_{s}C_{A}^{0}}}{1+{exp}^{\left[\frac{F}{RT}(E-E{}^{\circ})\frac{FcCOOH}{FcCOOH^{+}}\right]}}\;$$

A plot $\frac{i_{k}}{i_{p}^{0}}$ vs. $1+{exp}^{\left[\frac{F}{RT}(E-E{}^{\circ})\frac{FcCOOH}{FcCOOH^{+}}\right]}$ has a slope equal to $2.24\sqrt{\frac{RT}{Fv}2k_{s}C_{A}^{0}}$, which allowed us to calculate the rate constant. At 298.25 K, a k_s_ value of 2 × 10^5^ ± 5.0 × 10^3^ L/(mol·s) was obtained.

### 2.4. Calculation of Kinetic Activation Parameters

**Figure 4:**
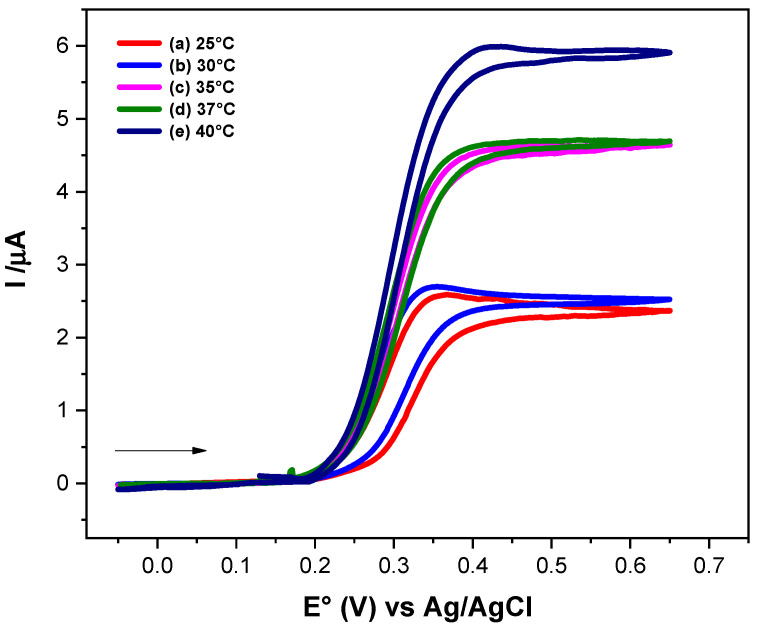
Cyclic voltammograms of 1 × 10^−3^ M Fc-COOH, in PBS 0.1 M (pH = 7.2), in the presence of 0.05 M glucose and 0.17 mM GOD enzyme at scanning rate of 1 mV/s, at different temperatures.

**Figure 5:**
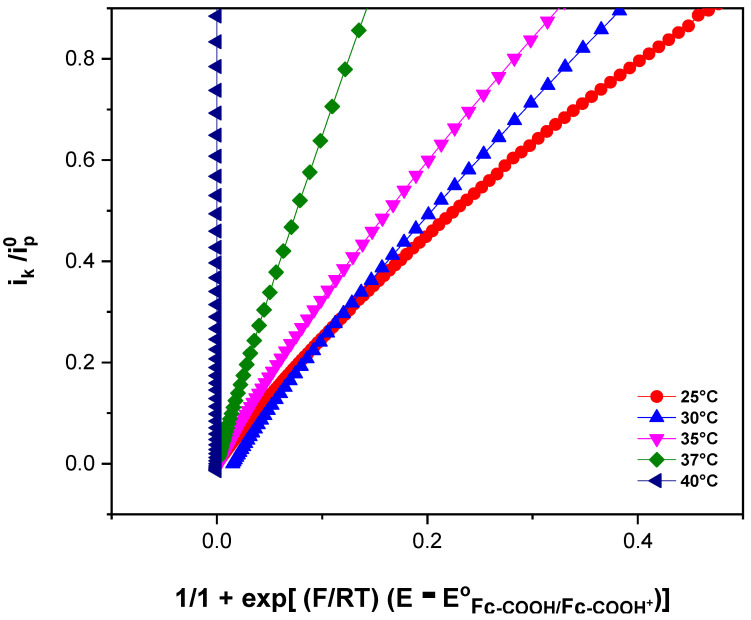
FOWA of 1 × 10^−3^ M Fc-COOH in PBS 0.1 M (pH = 7.2) in the presence of 0.05 M glucose and 0.17 mM GOD enzyme at a scanning rate of 1 mV/s.

Figure 4 shows the electrochemical responses of Fc-COOH in the presence of 0.17 mM GOD and 0.05 M D-glucose at different temperatures, recorded at a scan rate of 1 mV/s. An increase in the catalytic current is observed as the temperature rises, indicating an enhancement of the electron transfer kinetics. To determinate the homogeneous electron transfer rate constant (k_s_) at each temperature and to evaluate the corresponding activation parameters, FOWA was performed (Figure 5).

**Figure 6:**
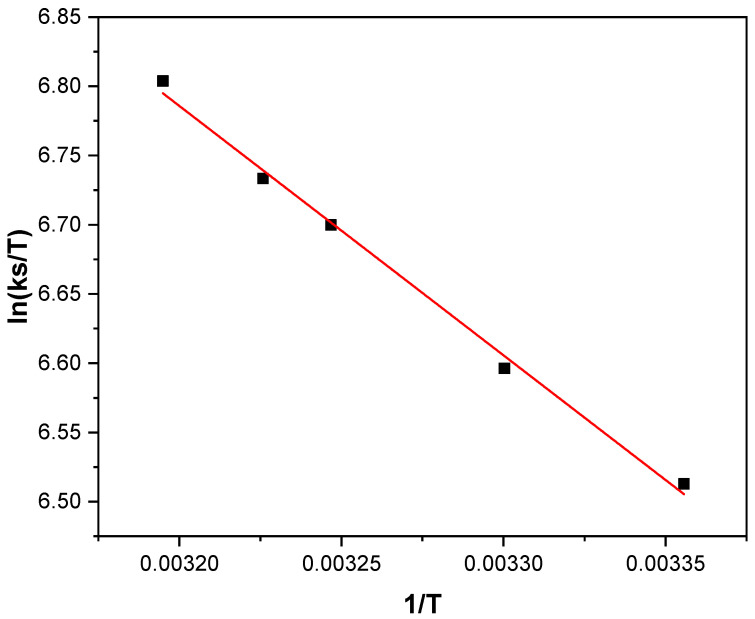
ln(k_s_/T) vs. 1/T plot of 1 × 10^−3^ M Fc-COOH in phosphate buffer (pH = 7.2), k*_s_* values obtained from FOWA in the presence of 0.05 M glucose and 0.17 mM GOD enzyme using a scan rate 1mV/s at different temperatures.

According to the transition state theory, the activation parameters can be obtained from a plot of ln(k_s_/T) vs. (1/T) (Figure 6) following Equation (13). Although this relationship was originally developed for gas phase reactions, it has been successfully applied to describe electron transfer processes in both metal complexes and proteins in solution [32,33,34,35]. The activation enthalpy $\left({{∆H}_{ET}}^{‡}\right)$ and the activation entropy $\left({{∆S}_{ET}}^{‡}\right)$ were derived from the slope and intercept of the linear fit, respectively.

The resulting ${{∆H}_{ET}}^{‡}$ and ${{∆S}_{ET}}^{‡}$ values indicate that the activated complex formed during the homogeneous electron transfer step exhibits a relatively low degree of disorder. This behavior suggests that the interaction between the redox mediator and the enzyme is sterically constrained and involves modes reorganization energy, which is consistent with a controlled molecular approach within the protein environment [36].

(13) $$\ln\left(\frac{k_{s}}{T}\right)=\ln\left(\frac{K_{B}}{h}\right)+\left(\frac{{{∆S}_{ET}}^{‡}}{RT}\;\right)-\left(\frac{{{∆H}_{ET}}^{‡}}{RT}\right)$$

Table 1 summarizes the equilibrium constant and thermodynamic parameters for the interaction between GOD and Fc-COOH, as well as the rate constant and activation parameters associated with the redox mediation process between GOD and Fc-COOH. The obtained k_s_ value (2.0 × 10^5^ L·mol^−1^·s^−1^) places Fc-COOH among the fastest homogeneous redox mediators reported, comparable to the high performance behavior of [OsCl(Him)(bpy)_2_]^+^ (2.81 × 10^5^ L/(mol·s) and [(η-MeC_5_H_4_)Mn(NO)(CN)_2_]Na (2.1 × 10^5^ L/(mol·s), and confirm the value for Fc-COOH obtained by the Nicholson and Shain method previously described (2.01 × 10^5^ L/(mol·s) [13,17,18,37]. This remarkable performance is noteworthy given the simplicity of the molecule, underscoring its efficiency as a homogeneous electron transfer mediator and supporting its suitability for bioelectrochemical applications.

Furthermore, the low activation enthalpy obtained (${{∆H}_{ET}}^{‡}$ = 14.9 kJ/mol) indicates minimal energetic requirements for electron transfer, whereas the strongly negative activation entropy $\left({{∆S}_{ET}}^{‡}\right)$ reflects the formation of a highly ordered transition state. The resulting activation free energy (${{∆G}_{ET}}^{‡}$ = 42.7 kJ/mol) falls within the expected range for biological electron transfer processes, reinforcing the mechanistic validity of the proposed system [38,39]. Notably, a lack of comparable data for GOD interacting with other redox mediators has been reported in the literature. As mentioned previously in Section 2.1, future work will focus on investigating the molecular interaction between GOD and other ferrocene derivatives in order to develop a comprehensive model that accounts for these effects on glucose biosensing performance.

### 2.5. Study of the GOD–Mediator Interaction by Circular Dichroism Spectroscopy

**Figure 7:**
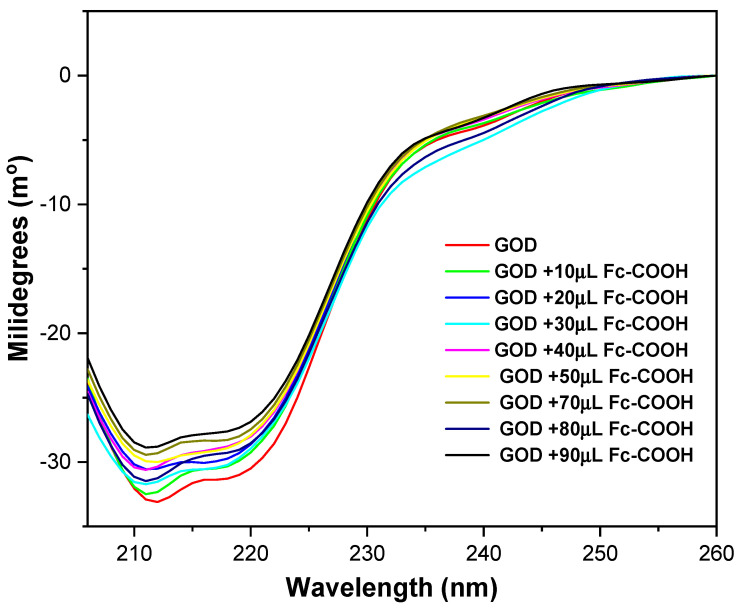
Circular dichroism spectra of 0.17 mM GOD enzyme in phosphate buffer (pH = 7.2), with additions of Fc-COOH at 25 °C.

After confirming the interaction between the enzyme and the mediator, circular dichroism (CD) spectroscopy was employed to evaluate whether GOD undergoes any conformational changes upon complex formation [40]. The CD spectrum of GOD shows two negative ellipticity bands at around 211 nm and 218 nm, which are characteristic of an a-helical secondary structure (Figure 7) [41]. These bands remained essentially unchanged after the addition of the redox mediator, indicating that the secondary structure of the enzyme is preserved. The minor variations detected are likely due to the intrinsic UV absorption of Fc-COOH, which can introduce slight interference in the CD measurements.

### 2.6. UV–Vis and Circular Dichroism Spectroelectrochemical Experiments

To assess potential structural or molecular changes occurring during the electron transfer process between GOD and Fc-COOH, UV–Vis and circular dichroism (CD) spectroelectrochemical measurements were performed. Figure S8 shows the UV–Vis spectra recorded using a platinum mesh as an optical transparent electrode (OTE). Upon application of a potential step to 400 mV, the Fe(II) transition ^1^A_1g_ → ^1^E_1g_ decreases in intensity, while a new absorption band at 625 nm, assigned to the Fe(III) transition ^2^E_g2_ → ^2^E_1g_, grows during electrolysis.

Additional spectroelectrochemical experiments carried out for Fc-COOH in the presence of 0.05 M glucose and 0.51 mM GOD reveal a continuous increase in the Fe(III) bands transition, ^2^E_g2_ → ^2^E_1g_, whereas the Fe(II) band remains nearly constant. This behavior confirms the occurrence of a homogeneous electron transfer between Fc-COOH^+^ and the reduced form of GOD.

**Figure 8:**
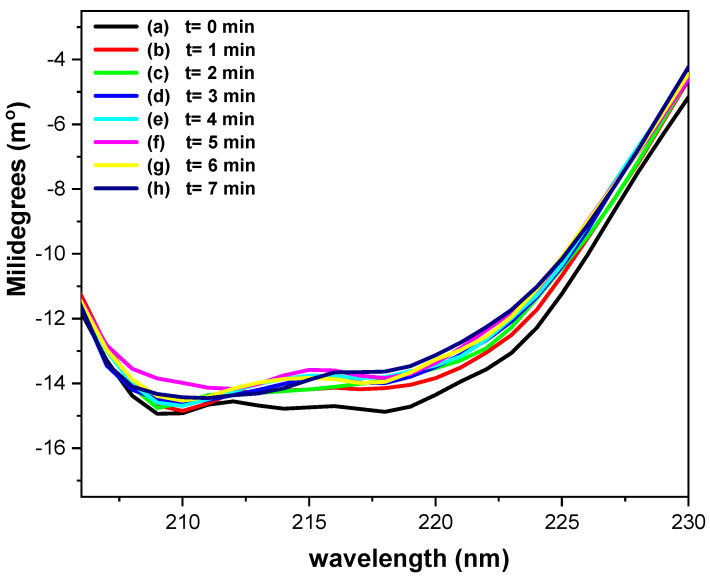
Circular dichroism spectroelectrochemical spectra of 0.17 mM GOD in phosphate buffer (pH = 7.2), using a platinum OTE, applying a potential at 400 mV vs. Ag/AgCl, each spectrum was acquired every minute.

Figure 8 shows the CD spectra of 0.17 mM GOD in phosphate buffer (pH = 7.2) recorded using a platinum OTE, while applying a potential of 400 mV vs. Ag/AgCl at one-minute intervals. Ellipticity changes at 218 nm provide insight into the α-helical content of the protein [42,43]. As illustrated in Figure 8 (a), before potential application the ellipticity, it is approximately 14.85 millidegrees. Upon applying a potential of 400 mV vs. Ag/AgCl, the a-helical content increases from roughly 5% (Figure 8, (b)) to nearly 10% over the course of the experiments (Figure 8, (b)–(h)). The ratio θ_218nm_/θ_211nm_, is used to evaluate the interaction between a-helices [40]; the value of 0.8–0.9 indicates the absence of interaction, whereas those near 1.0 ± 0.03 correspond to two-stranded coiled structure.

In the present CD spectroelectrochemical studies, the ratio values before (0.99) and during the electrolysis (≈0.95) remain within this range, suggesting no significant perturbation of a-helix interactions. These results suggest that the applied potential does not induce substantial alterations in the secondary structure of GOD during the electron transfer process.

**Figure 9:**
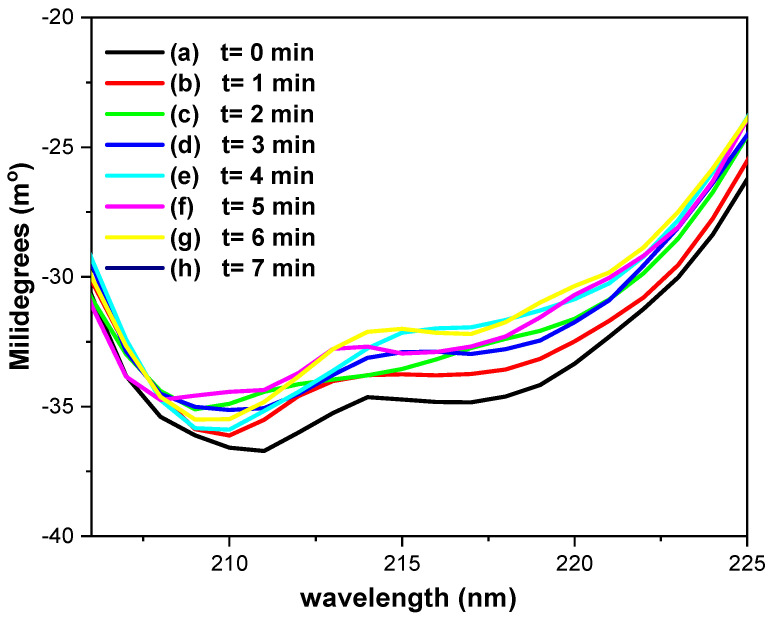
Circular dichroism spectroelectrochemical spectra of 0.17 mM GOD in phosphate buffer (pH = 7.2), acquired using a platinum OTE in the presence of 0.05 M glucose and 1 × 10^−3^ M Fc-COOH, and with a potential at 400 mV vs. Ag/AgCl, each spectrum was acquired every minute.

Figure 9 presents the CD spectroelectrochemical results for 0.17 mM GOD in the presence of Fc-COOH and glucose in phosphate buffer (pH = 7.2), recorded under the same experimental conditions. Relative to the initial CD spectrum (Figure 9, (a)), a decrease of no more than 10% in the a-helical content is observed during electrolysis (Figure 9, (b)–(h)). The ellipticity ratio θ_218nm_/θ_211nm_ decreases slightly from 0.94 (Figure 9, (a)) to 0.91 (Figure 9, (b)–(h)), which is a range consistent with non-interacting a-helices.

These observations demonstrate that, both in the absence and presence of Fc-COOH and glucose, the secondary structure of GOD remains essentially preserved throughout the electrolysis process.

## 3. Materials and Methods

### 3.1. Materials

All chemicals and solvents were used as received from Aldrich Chemical Company (St. Louis, MO, USA), Acros Organics (Geel, Belgium) and J.T. Baker (Radnor, PA, USA). Glucose Oxidase (GOD Ec 1.1.3.4 type II) from *Aspergillus niger* (molecular weight of 186,000) was purchased from Sigma Aldrich (St. Louis, MO, USA). The enzyme concentration was determined spectrophotometrically following a method previously reported in the literature [44].

### 3.2. Methods

#### 3.2.1. UV–Vis and Circular Dichroism Spectroscopy Studies

UV–Vis and circular dichroism (CD) measurements were carried out using a Biologic MOS-500 spectrophotometer over the 205–402 nm wavelength range, employing 0.1 cm optical path length quartz cuvette. Spectra of GOD (0.17 mM; 500 mL in PBS pH = 7.2) were recorded both in the absence and in the presence of successive additions (10mL) of a 1 mM ferrocene carboxylic acid (Fc-COOH) solution prepared in PBS (pH = 7.2). All measurements were conducted under temperature-controlled conditions using a Peltier unit. Spectra were corrected for dilution effects, and each measurement was carried out in triplicate. The interaction constant corresponds to the main value ± standard deviation.

#### 3.2.2. Electrochemical Studies

Cyclic voltammetry measurements were performed using a Biological SP-300 potentiostat/galvanostat. Solutions of 1 × 10^−3^ M ferrocene carboxylic acid (Fc-COOH) in 0.1M PBS (pH = 7.2) employed as supporting electrolyte. A thermostated, classical three-electrode electrochemical cell was used, consisting of a glassy carbon disk electrode (F = 3mm) as working electrode, a platinum wire as the auxiliary electrodes, and a Basi Ag/AgCl (3 M NaCl) reference electrode equipped with a Luggin capillary. Prior to each measurement, the solutions were purged with nitrogen for 5 min. The working electrode was cleaned by manual polishing with 1mm diamond powder, followed by rinsing with distilled water and brief sonication. The R_u_ was around 100 W. All measurements were carried out by triplicate at 25, 30, 35, 40, 45, 50, 55 and 60 °C.

#### 3.2.3. Determination of Homogeneous Electron Transfer Rate Constant (k_s_) for GOD Mediation

Cyclic voltammetry experiments were conducted using a Biologic SP-300 potentiostat/galvanostat. Measurements were performed in 1 × 10^−3^ M ferrocene carboxylic acid solutions prepared in 0.1 M phosphate buffer (pH = 7.2) containing 0.05 M glucose and 0.17 mM glucose oxidase (GOD), using a thermostated electrochemical cell. Prior to each experiment, the solutions were purged with nitrogen for 5 min. The working electrode was cleaned following the procedure detailed in the previous section. Measurements were recorded at 25, 30, 35, 40, 45, 50, 55, and 60 °C. All experiments were performed in triplicate, and the reported rate constant corresponded to average values with their associated standard deviation.

#### 3.2.4. UV–Vis and Circular Dichroism Spectroelectrochemical Studies

Spectroelectrochemical experiments were carried out using a Biologic SP-50 potentiostat/galvanostat coupled either to UV–Vis diode array spectrophotometer (Thermo-Scientific evolution array) or to a circular dichroism spectropolarimeter, (MOS-500 Biologic, Paris, France). The optical transparent electrode (OTE) cell consisted of a 1mm optical path length quartz cuvette fitted with an optically transparent platinum mesh serving as the working electrode. A platinum wire was used as the auxiliary electrode, and a Basi Ag/AgCl (3 M NaCl) electrode served as the reference. Prior to each measurement, the solutions were purged with nitrogen, and electrochemical and spectroscopic data were recorded simultaneously. For UV–Vis experiments, 1 × 10^−3^ M solutions of the redox mediator in PBS (pH = 7.2) were used. For CD-coupled spectroelectrochemistry, a 0.17 mM GOD solution in PBS (pH = 7.2) was analyzed in the absence and presence of 1 × 10^−3^ M ferrocene carboxylic acid and 0.05 M glucose. Due to the use of a diode array UV–Vis spectrophotometer and a scanning CD spectropolarimeter, UV–Vis spectra were collected every 20 s, whereas CD spectra were acquired at 1 min intervals.

## 4. Conclusions

The thermodynamic parameters ΔH_int_, ΔS_int,_ and ΔGi_nt_ obtained indicate a significant affinity between Fc-COOH and GOD, revealing an entropy-driven, hydrophobic interaction and a thermodynamically favorable spontaneous association. The thermodynamic parameters determined for Fc-COOH (ΔG°, ΔH°, and ΔS°) represent new findings for this redox mediator and suggest strong stabilization of the redox pair, which can be attributed to the small difference in solvation energy between its oxidized and reduced forms, which is consistent with an outer sphere electron transfer mechanism. The activation parameters ${{∆H}_{ET}}^{‡}$, ${{∆S}_{ET}}^{‡}$, and ${{∆G}_{ET}}^{‡}$ indicate minimal energetic requirements for electron transfer, the formation of a highly ordered transition state, and an activation free energy within the expected range for biological electron transfer processes. However, to the best of our knowledge, neither molecular interaction parameters (ΔH_int_, ΔS_int_, ΔGi_nt_) nor electron transfer activation parameters (${{∆H}_{ET}}^{‡}$, ${{∆S}_{ET}}^{‡}$, and ${{∆G}_{ET}}^{‡}$) for GOD interacting with other redox mediators have been reported in the literature, opening new avenues for research in this field. Future work will focus on investigating the molecular interaction between GOD and other ferrocene derivatives to propose a model that accounts for these effects on glucose biosensing performance. Moreover, using circular dichroism, UV–vis spectroscopy, and combined CD and UV–Vis spectroelectrochemical measurements, it was demonstrated for the first time that no significant structural changes occur in GOD during the electron transfer process, highlighting the value of coupling electrochemical and spectroscopic techniques.

## Figures and Tables

**Table 1:** Data of equilibrium constant (K_int_), thermodynamic parameters (ΔH_int_, ΔS_int_ and ΔG_int_) for the interaction of GOD with Fc-COOH, and rate constant (ks) with activation parameters (ΔH_ET_^‡^, ΔS_ET_^‡^ and ΔG_ET_^‡^) for the redox mediation (electron transfer) of GOD with Fc-COOH.

^a^ K_int_	^b^ ΔH_int_ J/mol	^b^ ΔS_int_ J/mol	^c^ ΔG_int_ J/mol	^d^ k_s_ L/(mol·s)	^e^ ΔH_ET_^‡^ kJ/mol	^e^ ΔS_ET_^‡^ kJ/(mol·K)	^f^ ΔG_ET_^‡^ kJ/mol
125 ± 10	1770 ± 140	132 ± 15	−31.04	2.0 × 10^5^ ± 5 × 10^3^	14.9 ± 2	−93 ± 10	42.7

^a^ Calculated at 25 °C and using Equation (5). ^b^ Calculated with Equation (6). ^c^ Calculated with ΔG_int_ = ΔH_int_ − TΔS_int_ at 25 °C. ^d^ Calculated at 25 °C with Equation (12). ^e^ Calculated at 25 °C with Equation (13). ^f^ Calculated with ΔG_ET_^‡^ = ΔH_ET_^‡^ − TΔS_ET_^‡^, at 25 °C. All data are reported at pH = 7.2.

## Data Availability

The data presented in this study are available in the article.

## Supplementary Materials

The following supporting information can be downloaded at https://www.mdpi.com/article/10.3390/molecules31010102/s1, Figure S1: Electronic spectra of GOD in phosphate buffer (pH 7.2). Figure S2: Electronic spectra of Fc-COOH in phosphate buffer (pH 7.2). Figure S3: Van’t Hoff plot for the interaction between Fc-COOH and GOD in phosphate buffer (pH 7.2). Figure S4: UV–Vis spectra of 1.7 mM BSA in PBS 0.1 M (pH 7.2) in the presence of Fc-COOH 1 mM at 25 °C. Figure S5: Cyclic voltammogram of 1 × 10^−3^ M Fc-COOH, in PBS (pH 7.2), at scan rates values of 1, 2, 5, 10, 15, 20, 25, 50, 75, 100, 250, 500, and 750 mV/s. Figure S6: Cyclic voltammogram of 1 × 10^−3^ M Fc-COOH, in PBS (pH 7.2), at 100 mV/s, using a temperature range from 25 to 50 °C. Figure S7: E° vs. T plot for Fc-COOH, in PBS (pH 7.2), at 100 mV/s, using a temperature range from 25 to 50 °C. Figure S8: Spectro electrochemical response UV–Vis region of 1 × 10^−3^ M Fc-COOH in PBS (pH 7.2), using an OTTLE, applying a constant potential value of 400 mV vs. Ag/AgCl. Spectra were acquired every 20 min.

## Abbreviations

The following abbreviations are used in this manuscript:

GOD	Glucose oxidase
FAD	Flavin Adenine Dinucleotide
FADH_2_	Reduced Flavin Adenine Dinucleotide
Fc-COOH	Ferrocene carboxylic acid
