# Supplementary material for: Thermodynamic, Kinetic, and UV–Vis/CD Spectroelectrochemical Studies on Interaction and Electron Transfer Between Glucose Oxidase and Ferrocene Carboxylic Acid
Source: Molecules. 2025 Dec 26;31(1):102. doi: 10.3390/molecules31010102 (PMC12786606; doi:10.3390/molecules31010102)
Supplement: Supplementary file 1 [file molecules-31-00102-s001.zip › molecules-4040005-supplementary.pdf]

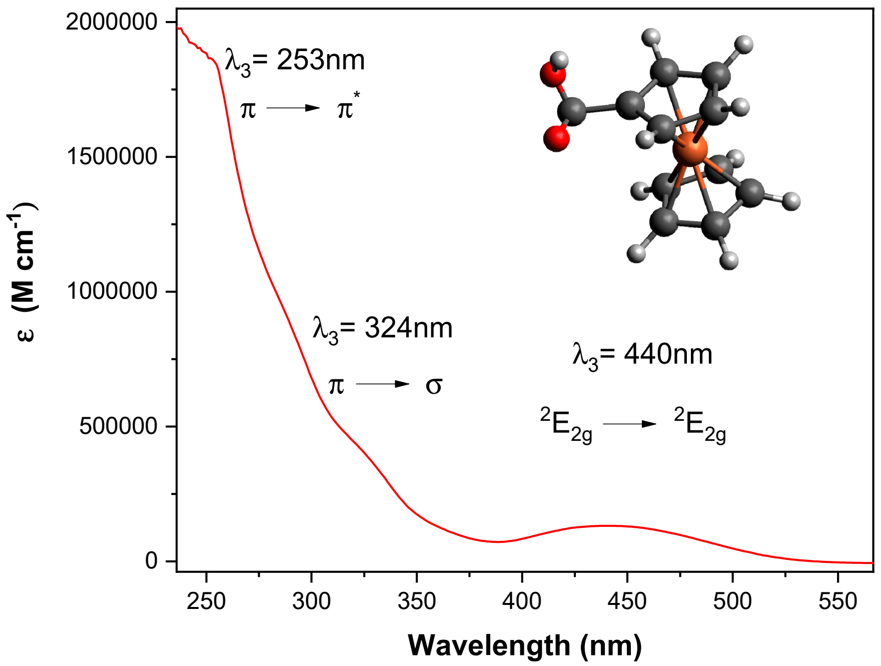

Figure S1. Electronic spectrum of Fc-COOH in phosphate buffer (pH 7.2).

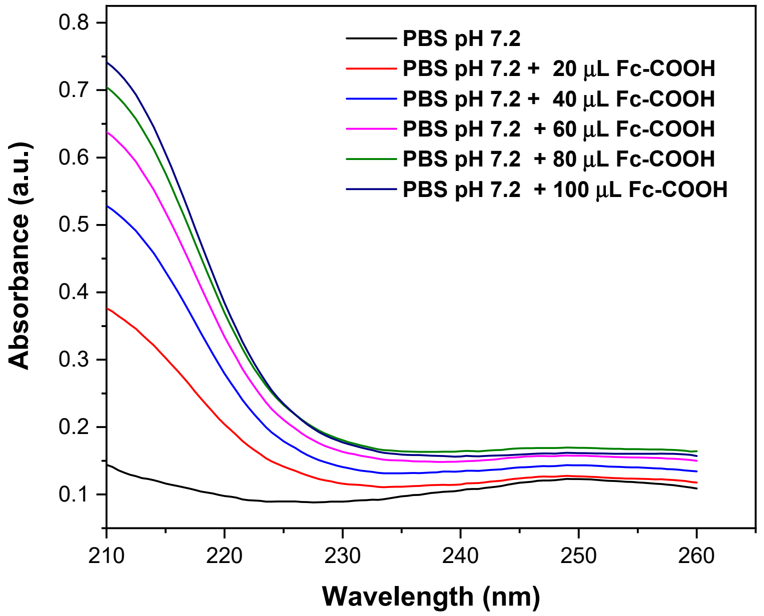

Figure S2. UV- vis spectrum of phosphate buffer (pH 7.2) in the presence of Fc-COOH 1mM at 25 °C.

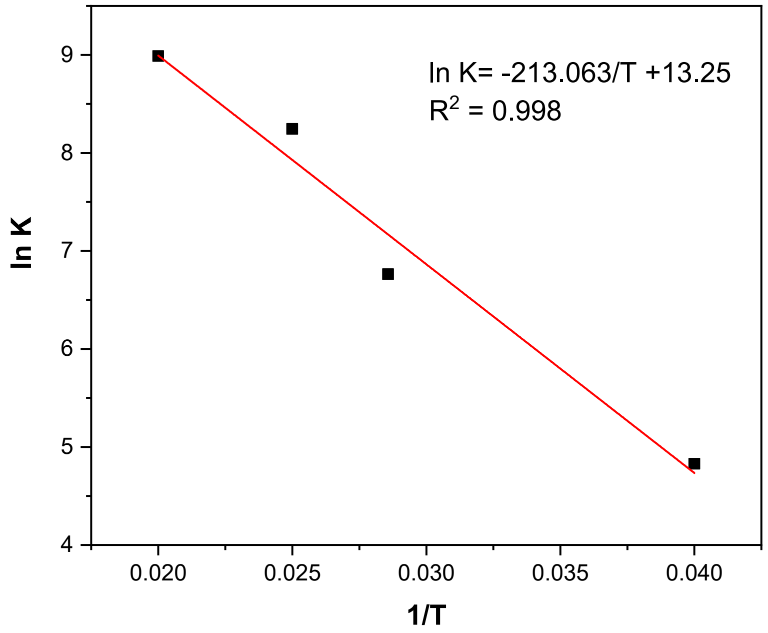

Figure S3. Van´t Hoff plot for the interaction between Fc-COOH and GOD in phosphate buffer (pH 7.2).

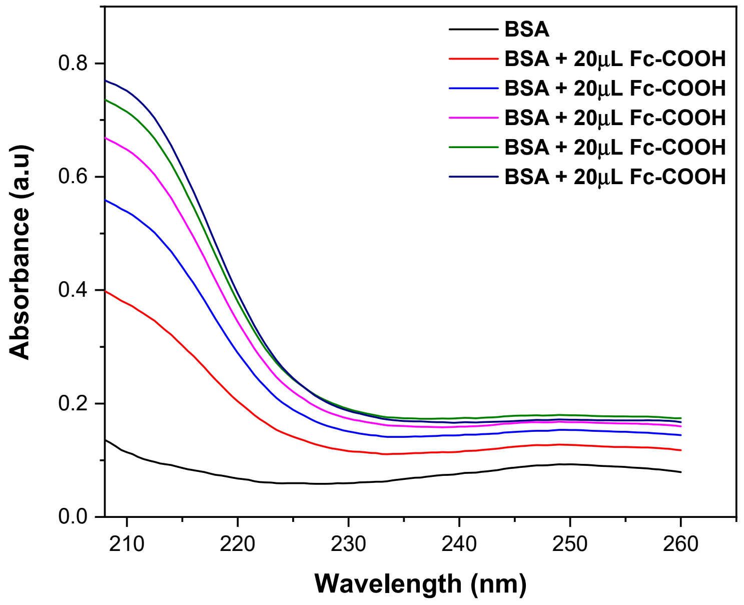

Figure S4. UV-vis spectra of 1.7 μM BSA in in PBS 0.1M (pH 7.2) in the presence of Fc-COOH 1mM at 25 °C.

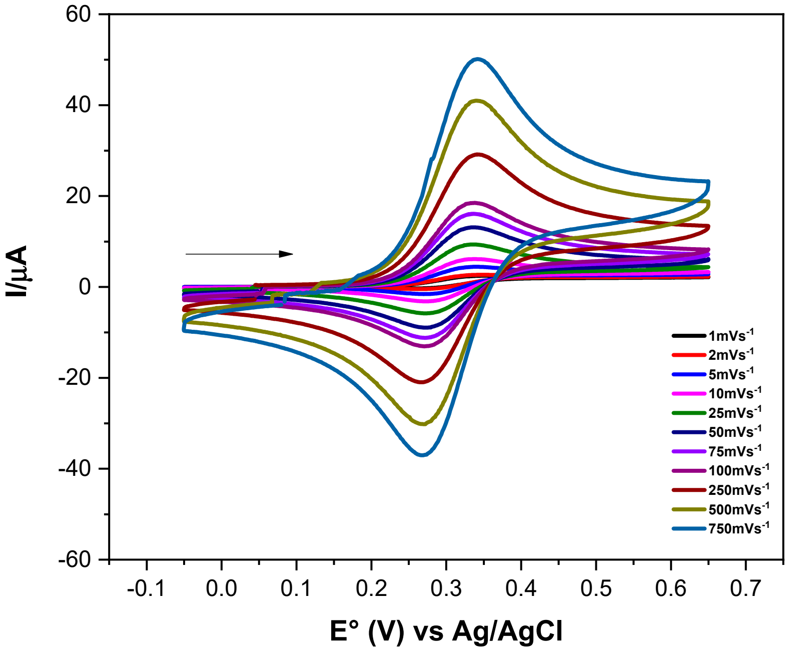

Figure S5. Cyclic voltammogram of 1×10^{-3} M Fc-COOH, in PBS (pH 7.2), at scan rates values of 1, 2, 5, 10, 25, 50, 75, 100, 250, 500, and 750 mV/s.

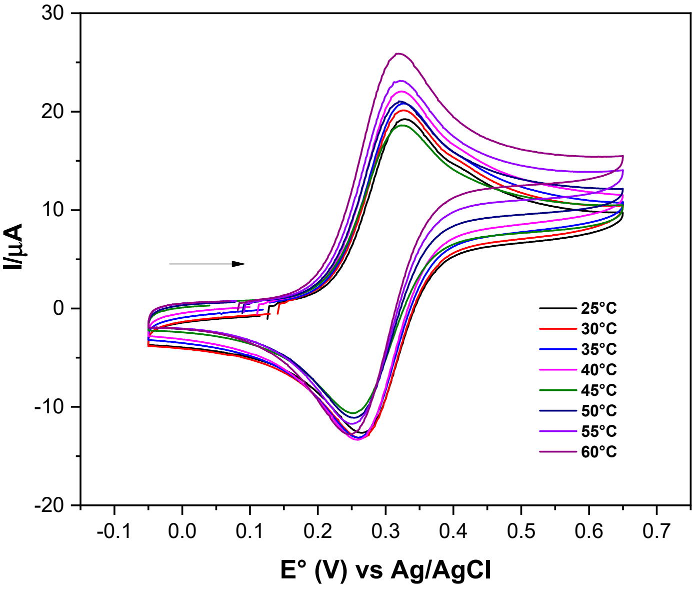

Figure S6. Cyclic voltammogram of 1×10^{-3} M Fc-COOH, in PBS (pH 7.2), at 100 mV/s, using a temperature range from 25 to 50 °C.

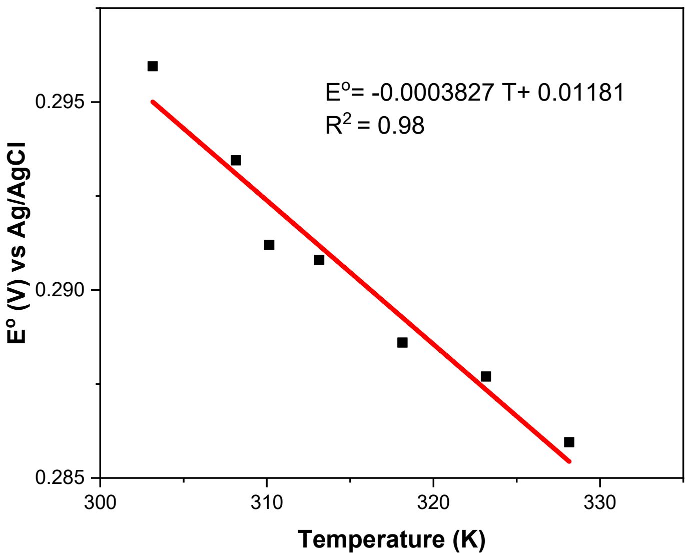

Figure S7. E º vs T plot for Fc-COOH, in BPS (pH 7.2), at 100 mV/s, using an experimental temperature range from 25 to 50 °C, converted into Kelvin.

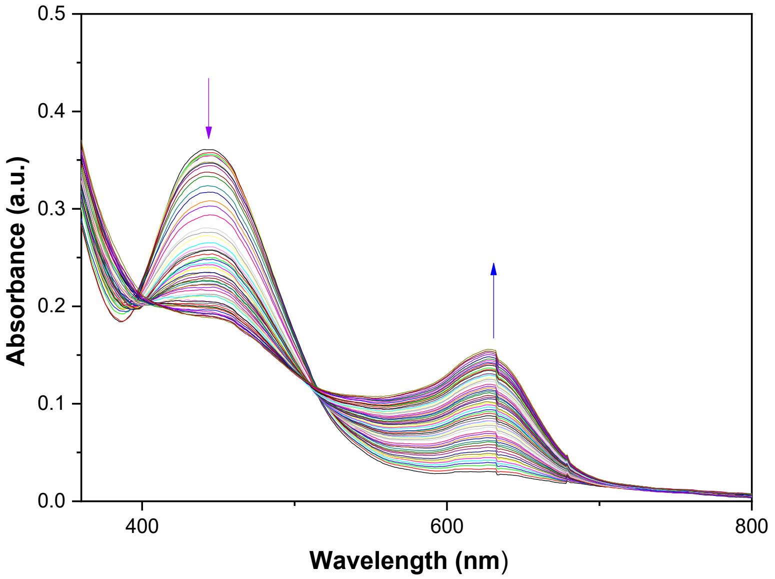

Figure S8. UV-Vis spectroelectrochemical response of 1×10^{-3} M Fc-COOH in PBS m f (pH 7.2), using an OTTLE, applying a constant potential value of 400 mV vs Ag/AgCl vs Ag/AgCl. Spectra were acquired every 20 seconds for 20 minutes.
